# Supplementary material for: KinConfBench: A Curated Benchmark for Cofolding Models on Kinase Conformational States
Source: bioRxiv. 2026 Apr 10:2026.04.07.716788. Preprint. [Version 1] doi: 10.64898/2026.04.07.716788 (PMC13082063; doi:10.64898/2026.04.07.716788)
Supplement: Supplement 1 [file media-1.pdf]

Supplementary Information for KinConfBench: A Curated Benchmark for Cofolding Models on Kinase Conformational States

[1]Kenneth S. Pitzer Theory Center and Department of Chemistry, [2]Department of Bioengineering, [3]Department of Chemical and Biomolecular Engineering, University of California, Berkeley, CA, 94720 USA
† corresponding authors: kysun@berkeley.edu; thg@berkeley.edu

S1 Additional Methodology Details **2**

S2 Supplementary Tables **4**

S3 Supplementary Figures **6**

S1 Additional Methodology Details

Kinase Group Classifications. To categorize the structural and functional diversity of the kinome, we utilize the standard Manning classification system [1]. The primary groups are defined as follows.

- **AGC**: Proteins including PKA, PKG, and PKC families.
- **CAMK**: Calcium/calmodulin-dependent protein kinases.
- **CK1**: Casein kinase 1 and closely related isoforms.
- **CMGC**: Proteins including CDK, MAPK, GSK3, and CLK families.
- **STE**: Homologs of yeast Sterile 7, Sterile 11, and Sterile 20 kinases.
- **TYR**: Tyrosine kinases.
- **TKL**: Tyrosine kinase–like proteins that are serine-threonine kinases.
- **OTHER**: Other kinases that don't fall into above classification.

Details of KinCoRe Annotations. Introduced by Modi and Dunbrack[2], a kinase conformational label has the following eight annotations:

- **Spatial Label:** Describes the position and orientation of the DFG motif as DFGin, DFGout, DFGinter, or None when key residues are not correctly mapped.
- **Dihedral Label:** Captures the backbone and side chain dihedral angles of the X-DFG motif by using the Ramachandran region annotation (A, B, L, and E) for the X, D, and F residues and the DFG-Phe χ_1 rotamer (minus = $-60°$, plus = $+60°$, and trans = $180°$). These labels include BLAminus, BLBplus, ABAminus, BBAminus, BLBtrans, BLBminus, BLAplus, BABtrans, or None when key residues are not correctly mapped.
- **αC-helix Label:** Indicates the placement of the αC-helix as either Chelix-in, Chelix-out, or None when key residues are not correctly mapped.
- **Salt Bridge Label:** Denotes the formation of the conserved salt bridge as SaltBr-in, SaltBr-out, or None when key residues are not correctly mapped.
- **N-term. Act. Loop Label:** Classifies the N-terminal region of the activation loop as ActLoopNT-in, ActLoopNT-out, or None when key residues are not correctly mapped.
- **C-term. Act. Loop Label:** Classifies the C-terminal region of the activation loop as ActLoopCT-in, ActLoopCT-out, or None when key residues are not correctly mapped.
- **Spine Label:** Indicates the assembly state of the regulatory spine as Spine-in or Spine-out, or None when key residues are not correctly mapped.
- **Ligand Type:** Categorizes the bound ligand into Type 1, Type 1.5_Front, Type 1.5_Back, Type 2, Type 3, Allosteric, and No_ligand.

Details of Metrics for Diversity Analysis.

- `lys_glu_distance`: Tracks the separation between the conserved β3-Lysine and αC-Glutamate, critical for evaluating αC-helix packing.
- `saltbridge_distance`: Monitors the formation and structural stability of the canonical salt bridge network.
- `spine_distance`: Measures the spatial arrangement of the hydrophobic residues comprising the regulatory spine, indicating its assembly state.
- `dfg_hrd_distance`: Evaluates N-terminal activation-loop coupling by measuring the spatial separation between the DFG and catalytic HRD motifs.
- `asp_phi`: Captures the ϕ backbone dihedral angle of the DFG Aspartate residue.
- `asp_psi`: Captures the ψ backbone dihedral angle of the DFG Aspartate residue.
- `asp_chi1`: Describes the primary side-chain torsion (χ_1) of the DFG Aspartate.
- `asp_chi2`: Describes the secondary side-chain torsion (χ_2) of the DFG Aspartate. This is treated with twofold ("π") symmetry so that flipped carboxylate placements are not penalized as opposite phases on the circle.
- `phe_phi`: Captures the ϕ backbone dihedral angle of the DFG Phenylalanine residue.
- `phe_psi`: Captures the ψ backbone dihedral angle of the DFG Phenylalanine residue.
- `phe_chi1`: Describes the primary side-chain torsion (χ_1) of the DFG Phenylalanine.
- `phe_chi2`: Describes the secondary side-chain torsion (χ_2) of the DFG Phenylalanine. Like the Aspartate, this is treated with twofold ("π") symmetry so that flipped aromatic substituent placements are not counted as opposite phases.

S2 Supplementary Tables

Table S1: **Percentage of KinCoRe spatial and dihedral label combinations for human kinases.** The definitions of Spatial and Dihedral Labels are defined in Section S1. From the KinCoRe server PDB inventory (https://dunbrack.fccc.edu/kincore/home) and for KinConfBench (KCB) reference chains which focuses on a subset of high quality samples and filters out None labels during selection.

Spatial Label	Dihedral Label	All PDB	KinConfBench
DFGin	BLAminus	6806	1430
DFGin	BLAplus	316	27
DFGin	ABAminus	993	183
DFGin	BLBminus	433	96
DFGin	BLBplus	1008	238
DFGin	BLBtrans	255	102
DFGinter	BABtrans	26	5
DFGout	BBAminus	677	144
DFGin	None	836	0
DFGinter	None	224	0
DFGout	None	365	0
None	None	413	0

Table S2: **Distribution of KinCoRe spatial and dihedral labels for KinConfBench (KCB) and cofolding model predictions.** KCB percentages are derived from the 2,225-chain set described in the Results. Percentages for Boltz-2 ($n = 28,400$), Chai-1 ($n = 28,399$), and Protenix ($n = 28,400$) are calculated across 1,420 distinct kinase systems. * The small percentage of "None" labels results from residue mismatches during the KinCoRe labeling process.

Spatial	Dihedral	KCB (%)	Boltz-2 (%)	Chai-1 (%)	Protenix (%)
DFGin	BLAminus	64.3	65.8	77.5	65.1
DFGin	BLAplus	1.2	1.1	1.2	1.5
DFGin	ABAminus	8.2	3.9	4.8	3.3
DFGin	BLBminus	4.3	3.0	2.0	3.4
DFGin	BLBplus	10.7	8.0	8.5	8.3
DFGin	BLBtrans	4.6	11.6	0.1	11.7
DFGinter	BABtrans	0.2	0.0	0.0	0.0
DFGout	BBAminus	6.5	5.3	4.3	5.4
DFGin	None*	0.0	0.4	0.4	0.2
DFGinter	None*	0.0	0.8	0.6	0.1
DFGout	None*	0.0	0.1	0.5	0.1
None*	None*	0.0	0.0	0.1	1.0

Table S3: **Conformational (KinCoRe label) summary on 950 systems passing geometric filters.** Systems ≥1 correct: systems with at least one all-labels-correct prediction in top-20. Avg correct/system: mean KinCoRe-correct predictions per system (out of top-20).

Model	Systems ≥1 correct	Avg correct/system
Boltz-2	779 (82.0%)	14.2
Chai-1	724 (76.2%)	12.4
Protenix	776 (81.7%)	14.1

Table S4: **110 systems where all cofolding predictions fail to generate the correct conformation as the ground truth.** Each entry is a KinConfBench system key (`GENE_Name_Ligands`).

System identifiers				
ABL1_HUMAN_66K	ABL1_HUMAN_STI_1N1	ACK1_HUMAN_1G0	ACK1_HUMAN_DBQ	ACK1_HUMAN_LWX
ACK1_HUMAN_R7P	ACK1_HUMAN_T74	ACK1_HUMAN_T95	ACK1_HUMAN_WTP	ALK_HUMAN_25J
ALK_HUMAN_6YL	ALK_HUMAN_AWJ	ALK_HUMAN_HKJ	ALK_HUMAN_J3Y	AURKA_HUMAN_9YQ
AURKA_HUMAN_X6D	BRAF_HUMAN_5I4	BRAF_HUMAN_FP3	BRAF_HUMAN_P06	BTK_HUMAN_YDA
CDK1_HUMAN_1QK	CDK2_HUMAN_02Z	CDK2_HUMAN_06Z	CDK2_HUMAN_09Z	CDK2_HUMAN_0S0
CDK2_HUMAN_106	CDK2_HUMAN_20K	CDK2_HUMAN_26Z	CDK2_HUMAN_A1A1H	CDK2_HUMAN_A1D6S
CDK2_HUMAN_A27	CDK2_HUMAN_DTQ	CDK2_HUMAN_ES4	CDK2_HUMAN_JWS	CDK2_HUMAN_LS1
CDK2_HUMAN_LS3	CDK2_HUMAN_LS4	CDK2_HUMAN_RON	CDK2_HUMAN_SU9	CDK2_HUMAN_WQ6
CDK2_HUMAN_X02	CDK2_HUMAN_X06	CDK2_HUMAN_X19	CDK2_HUMAN_X35	CDK2_HUMAN_X36
CDK2_HUMAN_X3A	CDK2_HUMAN_X40	CDK2_HUMAN_X42	CDK2_HUMAN_X43	CDK2_HUMAN_X44
CDK2_HUMAN_X62	CDK2_HUMAN_X6B	CDK2_HUMAN_Y8L	CDK2_HUMAN_Z19	CDK2_HUMAN_Z63
CDK2_HUMAN_Z71	CDK5_HUMAN_65L	CDK6_HUMAN_24V	CDK6_HUMAN_AP9	CDK6_HUMAN_LQQ
CDK7_HUMAN_I73	CDK7_HUMAN_WZ8	CDK8_HUMAN_C1I	CHK1_HUMAN_306	CHK1_HUMAN_373
CHK1_HUMAN_76A	CHK1_HUMAN_H0K	CHK1_HUMAN_YM6	CLK1_HUMAN_Q7K	CLK1_HUMAN_WAZ
DAPK1_HUMAN_BD4	DAPK1_HUMAN_LU2	DAPK1_HUMAN_PIT	DAPK1_HUMAN_STU	DAPK3_HUMAN_4RB
DCLK1_HUMAN_XBD	EPHA2_HUMAN_DXK	EPHA2_HUMAN_L66	EPHA2_HUMAN_QRD	EPHA2_HUMAN_QRR
EPHA2_HUMAN_WT3	IGF1R_HUMAN_PDR	JAK3_HUMAN_79T	KC1D_HUMAN_AUE	KCC2D_HUMAN_K88
KSYK_HUMAN_1B6	KSYK_HUMAN_4MG	KSYK_HUMAN_685	KSYK_HUMAN_X7G	M3K5_HUMAN_NJV
M3K5_HUMAN_STU	M4K1_HUMAN_2WI	M4K1_HUMAN_A1APO	M4K1_HUMAN_YK1	MK01_HUMAN_2SH
MK01_HUMAN_33A	MK01_HUMAN_35X	MK01_HUMAN_362	MK01_HUMAN_5ID	MK01_HUMAN_F29
MK01_HUMAN_FRZ	MK07_HUMAN_R4L	MK14_HUMAN_GK1	SRPK1_HUMAN_RXZ	VRK2_HUMAN_7DZ
VRK2_HUMAN_KJD	WEE1_HUMAN_34W	WEE1_HUMAN_99J	WEE1_HUMAN_99M	WEE1_HUMAN_99V

S3 Supplementary Figures

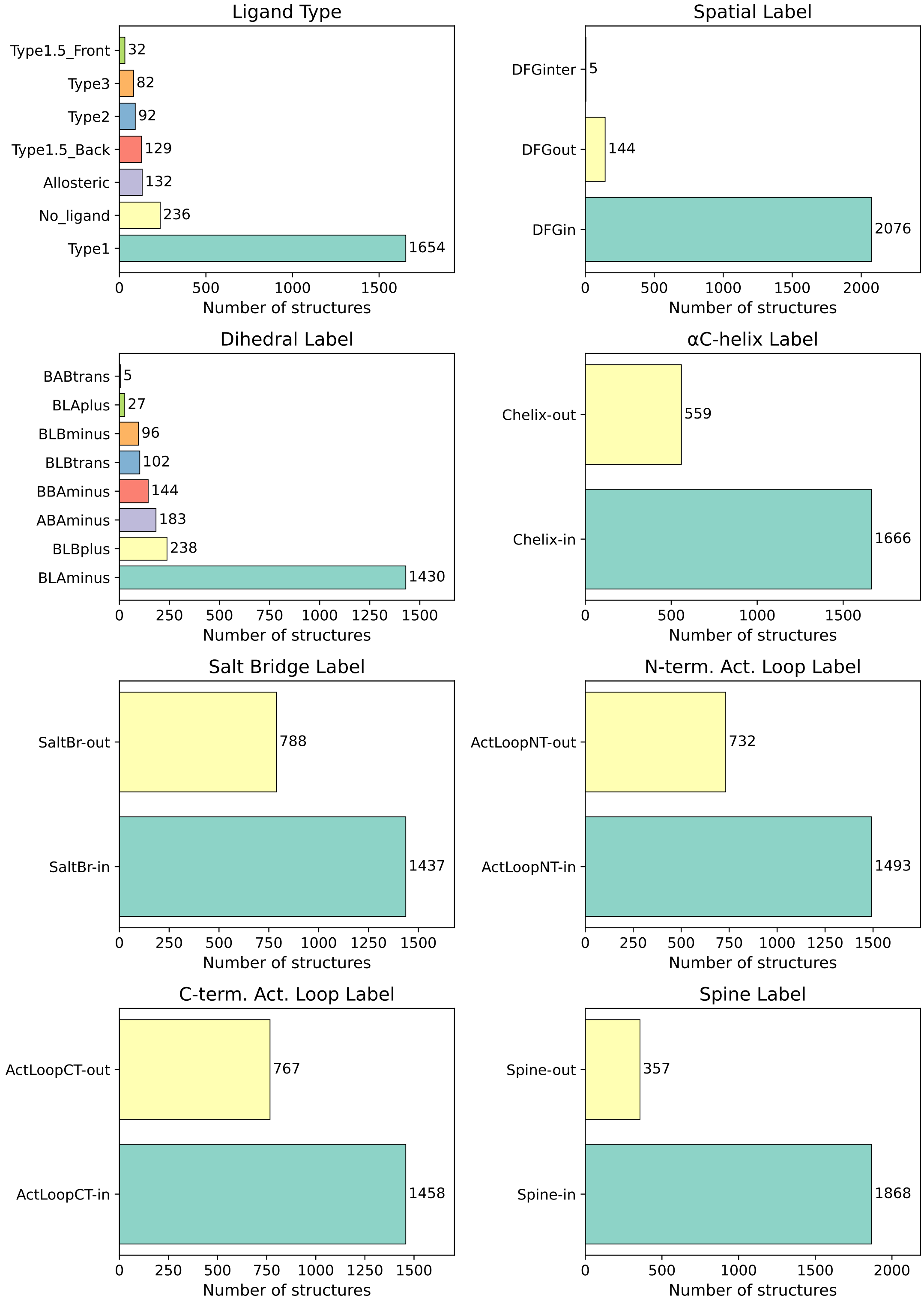

Figure S1: **KinConfBench curation and composition.** Summary of how the benchmark is built from PDB-derived kinase chains: counts by holo versus apo, single- versus multi-ligand complexes, redundancy filtering, and the distribution of entries across Manning kinase groups and ligand/chemotype categories. Together these panels document that the final benchmark is holo-heavy (reflecting the PDB) yet retains diverse genes and chemotypes, and that reported model statistics are computed on the curated non-redundant set rather than the raw PDB pull.

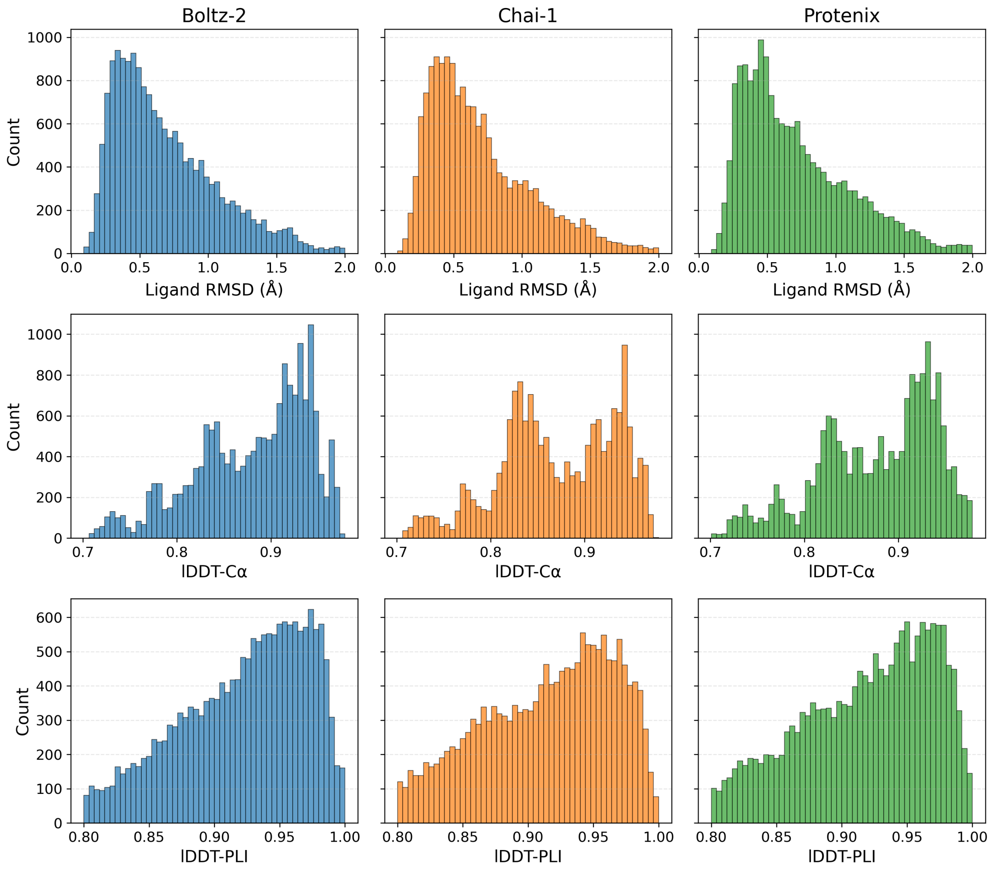

Figure S2: **Distribution of geometric metrics for cofolding predictions.** Distributions of ligand heavy-atom RMSD, lDDT-Cα, and lDDT-PLI for ensemble predictions that pass the benchmark geometric filters (lDDT-C$\alpha \geq 0.7$, lDDT-PLI ≥ 0.8, ligand RMSD < 2 Å). We have 950 protein-ligand kinase systems from Boltz-2 (Blue, 17,409 samples), Chai-1 (Orange, 16,471 samples), and Protenix (Green, 17,125 samples).

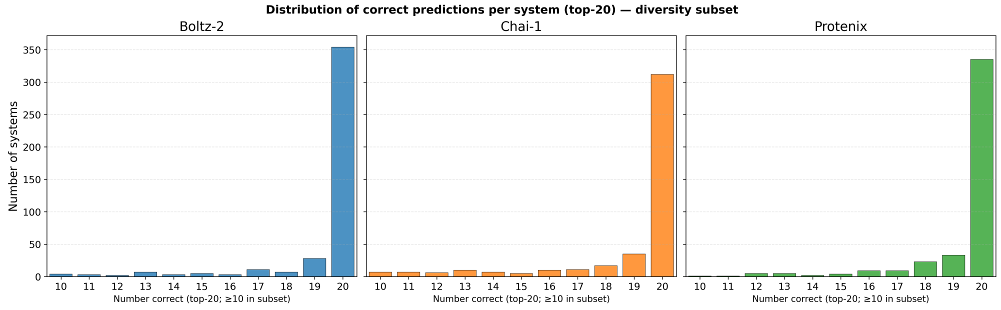

Figure S3: **Correct-prediction counts on the high-yield diversity subset.** Same quantity as in Figure 2(c), but restricted to 509 systems where Boltz-2, Chai-1, and Protenix each have at least ten all-labels-correct predictions in the top-20, the subset used for ensemble diversity analysis. The horizontal range is truncated to 10-20 so that differences in the upper tail (near-enumeration of the correct state) are visible. A single-row layout with a shared y-axis facilitates direct comparison of how densely each model covers the correct label manifold when it is readily accessible.

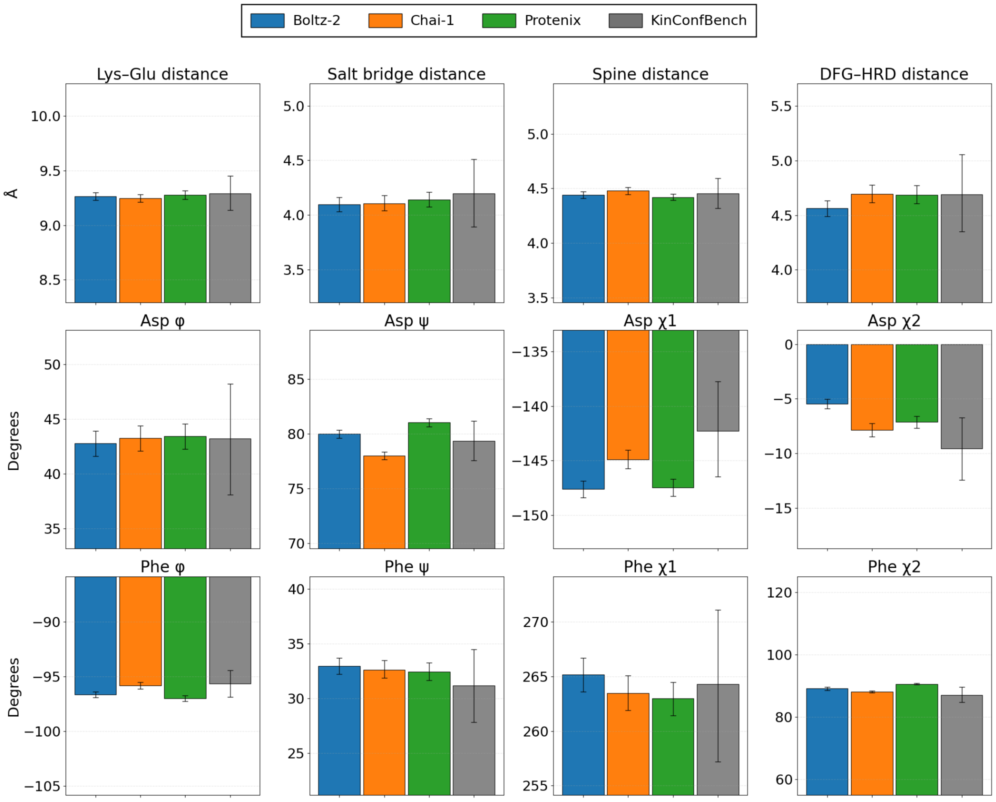

Figure S4: **Mean of key distance and angle metrics for cofolding predictions and KinConfBench.** The plot displays mean values for Boltz-2 (blue), Chai-1 (orange), and Protenix (green) predictions, with KinConfBench PDB reference values provided in gray for comparison. Error bars are bootstrap 95% confidence intervals for each pooled mean.

References

[1] Manning G, Whyte DB, Martinez R, Hunter T, Sudarsanam S. The Protein Kinase Complement of the Human Genome. Science. 2002 Dec;298(5600):1912-34. Available from: https://www.science.org/doi/10.1126/science.1075762.

[2] Modi V, Dunbrack RL. Kincore: a web resource for structural classification of protein kinases and their inhibitors. Nucleic Acids Research. 2022 Jan;50(D1):D654-64. Available from: https://academic.oup.com/nar/article/50/D1/D654/6395339.
